# Network pharmacology and transcriptomics reveal the mechanisms of FFBZL in the treatment of oral squamous cell carcinoma

**DOI:** 10.3389/fphar.2024.1405596

**Published:** 2024-09-11

**Authors:** Shiyang Zhao, Shudong Xiao, Wanting Wang, Xinyue Dong, Xichen Liu, Qingsen Wang, Yourong Jiang, Wen Wu

**Affiliations:** Department of Stomatology, Jining Medical University, Shandong, China

**Keywords:** FFBZL, oral squamous cell carcinoma, apoptosis, PI3K-Akt signaling pathway, network pharmacology

## Abstract

**Background:**

FFBZL is composed of three herbs: *Scutellaria barbata D. Don* (SBD), *Astragali Radix* (AR), and *Ligusticum chuanxiong Hort* (CX). FFBZL has been reported to be effective in the treatment of oral squamous cell carcinoma (OSCC). However, the molecular mechanism involved remains unclear. Based on network pharmacology combined with bioinformatics and molecular docking, the effect and molecular mechanism of action of FFBZL in treating OSCC were explored.

**Materials and methods:**

This study employed an integrated approach using various databases and literature sources to identify the effective components of FFBZL, with a specific emphasis on screening active ingredients that align with traditional Chinese medicine principles. The TCMSP, ETCM, and SymMap databases were utilized to collect information on the active constituents and targets of FFBZL, while the PharmMapper database was used to predict targets. Key components were selected based on the degree value of the ‘active component−target’ network. Transcriptome data for OSCC samples were obtained from the TCGA and GEO databases. Differential gene expression analysis was conducted to identify targets associated with OSCC, and these targets were subsequently aligned with targets of the effective components of FFBZL to identify common targets. Subsequently, the STRING database was utilized to construct a protein‒protein interaction (PPI) network of these common targets, which was subsequently visualized using Cytoscape. Next, 71 targets were rescreened using the PPI network, and GO and KEGG enrichment analyses were performed; the PI3K-Akt signaling pathway was the top-ranking pathway related to cell apoptosis. Next, the expression of 19 genes enriched in the PI3K-Akt signaling pathway was analyzed using OSCC transcriptome data from the TCGA and GEO databases. The targets were subsequently mapped to the PI3K-Akt signaling pathway using the KEGG database, and the GSEA algorithm was used to assess the overall expression trend of the genes in this pathway. The 71 common targets were subsequently imported into the STRING database and visualized using Cytoscape. The DEGREE and MCC algorithms were used to select the corresponding targets within the PPI network. The intersection of these targets and the 19 targets mapped to the PI3K-Akt signaling pathway led to the identification of 6 key targets associated with cell apoptosis: GSK3B, PIK3CA, FN1, MET, SPP1, and MAPK3. Subsequently, the UALCAN database was utilized to analyze the expression levels and survival associations of the key genes related to cell apoptosis, and the transcriptome data from the GEO database were used to assess the correlations among the 6 key genes. Finally, molecular docking studies were conducted to explore the relationships between these targets and the active components with predicted associations.

**Results:**

This study identified six key components of FFBZL (quercetin, wogonin, carthamidin, scutellarein, senkyunolide K and astragalosidei: astragaloside I) as well as 820 potential target genes of these components. Intersection of these targets with those related to OSCC yielded 151 common targets. GO and KEGG enrichment analyses revealed that most of the top-ranked functions and pathways were associated with apoptosis, with the PI3K-Akt signaling pathway playing a critical role. Transcriptome analysis of data from the TCGA and GEO databases indicated that the genes enriched in the PI3K-Akt signaling pathway were strongly upregulated, and the GSEA algorithm indicated an overall upregulation trend for the PI3K-Akt signaling pathway. By intersecting the targets with the 19 genes mapped to the PI3K-Akt signaling pathway using the DEGREE and MCC algorithms, six key targets related to cell apoptosis were identified. The mRNA and protein expression levels of most these targets in head and neck squamous cell carcinoma were higher than those in normal tissues. Survival analysis revealed that low expression of SPP1 and FN1 was associated with increased patient survival time. Additionally, the molecular docking results indicated strong binding potential between the six identified key components and the six key targets.

## 1 Introduction

Oral cancer is one of the most prevalent malignant tumors in the field of oral and maxillofacial surgery, and more than 90% of cases involve oral squamous cell carcinoma (OSCC). In 2020, 377,713 new cases and 177,757 related deaths were reported globally (Chamoli et al., 2021; Sung et al., 2021). The onset of OSCC is linked to overall health conditions, including personal living environment, dietary habits, smoking, alcohol consumption, betel nut chewing, and human papillomavirus infection (Kumar et al., 2016; Shibahara, 2017). Despite some progress made by traditional therapies, high mortality and incidence rates persist due to delayed diagnosis and ineffective treatment. Additionally, some patients experience various fatal adverse reactions during treatment. Therefore, it is crucial to develop new methods to eradicate oral cancer cells while minimizing adverse effects on normal cells (Ding et al., 2020). Traditional Chinese medicine has shown promise in addressing these challenges.

The heat-clearing and detoxifying Chinese herbal medicine *Scutellaria barbata D. Don* (SBD), also known as Hanxincao, belongs to the Lamiaceae family in the order Lamiales. The entire plant is used for medicinal purposes, is characterized by a pungent and neutral taste, and nontoxic. SBD contains a variety of active ingredients, including flavonoids, terpenoids, polysaccharides, organic acids, and steroids, which demonstrate effects such as heat-clearing effects, detoxification, promotion of blood circulation, resolution of blood stasis, diuresis, and reduction of swelling (Chen et al., 2020). Studies have indicated that SBD, either alone or in combination with other drugs, can be effective at treating various types of tumors, such as lung cancer (Liu et al., 2018), cervical cancer (Xue et al., 2022), colon cancer (Zhu et al., 2023), and liver cancer (Yang A. Y. et al., 2022). FFBZL has been shown to inhibit the proliferation of precancerous cells. However, due to the multicomponent, multitarget, and multipathway characteristics of FFBZL, its mechanism of action in inhibiting OSCC progression remains unclear. Therefore, there is an urgent need to establish a systematic research model to comprehensively elucidate the mechanism of action of FFBZL in inhibiting OSCC progression.

Bioinformatics is an interdisciplinary field in which complex relationships are studied within vast biological datasets; this type of analysis often relies on the internet as a medium and databases as a platform. Network pharmacology, on the other hand, is a pharmacological research method based on network and systems biology. Various biological data and analysis methods are integrated to reveal the interactions between drugs and targets, diseases, and biological networks. This emerging interdisciplinary field represents the convergence of big data and artificial intelligence in pharmacological research in the modern era. The combined application of bioinformatics and network pharmacology holds significant value in disease research and treatment. With the integration of diverse data resources and analysis methods, a deeper understanding of the molecular mechanisms of diseases can be achieved. This approach facilitates the discovery of new therapeutic targets and disease biomarkers, as well as the optimization of drug design and personalized treatment strategies, thereby driving progress in disease prevention and treatment (Li and Zhang, 2013). Therefore, this study is primarily based on the application of bioinformatics and network pharmacology to investigate the potential therapeutic targets and pharmacological mechanisms of various active components of FFBZL in combating OSCC. Furthermore, molecular docking techniques were employed to further validate the binding of active components with their respective targets. This research aimed to provide a basis for understanding the mechanisms of action of FFBZL in treating OSCC, offering new perspectives for the treatment of this disease.

## 2 Materials and methods

### 2.1 Collection and filtering of FFBZL active ingredients and targets

Through searches of the TCMSP, ETCM, and SymMap databases, the active ingredients and targets of FFBZL were obtained. These components were subsequently screened in accordance with the principles of traditional Chinese medicine to identify the effective components that met the specified criteria. The inclusion criteria for components were as follows: ① oral bioavailability (OB) ≥30 and ② drug likeness (DL) ≥0.18. For the active components without relevant target information, the chemical structures were retrieved from the PubChem database in standard SMILES format. The PharmMapper database (Wang et al., 2017) was subsequently used to predict the targets, and targets with a z’-score greater than 1 were selected (Prasad et al., 2021). After eliminating duplicates, gene names corresponding to the targets were obtained from the STRING database.

### 2.2 Determination of the key ingredients of FFBZL

The drugs, active components, and targets of FFBZL were imported into Cytoscape software to establish a “drug-ingredient-target” network. Subsequently, six critical active components of FFBZL were identified based on their degree values.

### 2.3 Downloading of OSCC data

We obtained RNA-seq data for the TCGA-HNSC cohort, from which 375 oral tissue samples were obtained (as of 13 October 2017); these samples included 343 oral squamous cell carcinoma samples and 32 normal oral tissue samples. Furthermore, we downloaded the GSE25099 dataset, which included information on 79 samples, consisting of 57 oral squamous cell carcinoma samples and 22 normal oral tissue samples.

### 2.4 Analysis of differentially expressed genes (DEGs)

We conducted differential expression analysis of the TCGA and GSE25099 samples using the R package “limma” (Ritchie et al., 2015). We applied |logFC| ≥ 1.0 and adj. *P* < 0.05 as the criteria for selecting DEGs and generated volcano plots using the “ggplot2” package.

### 2.5 Acquisition of common targets of FFBZL and OSCC

We utilized the jvenn tool to identify the intersection of DEGs from the TCGA and GSE25099 cohorts with the targets of FFBZL, resulting in the identification of common targets between FFBZL and OSCC. Subsequently, a Venn diagram was created to visualize these interactions. The common targets were subsequently input into the STRING database to construct a protein‒protein interaction network, which was visualized using Cytoscape software. The targets with a degree greater than the median were selected, and a network of “key components−common targets” of FFBZL was constructed using Cytoscape software.

### 2.6 Analysis of GO and KEGG enrichment

We employed DAVID to conduct GO and KEGG enrichment analyses of the common targets of FFBZL in OSCC. We selected the KEGG and WikiPathways databases for KEGG pathway clustering enrichment of the shared targets. Furthermore, we performed GO enrichment analysis of the biological process (BP), molecular function (MF), and cellular components (CC). A significance threshold of *P* < 0.05 was used for the selection of the top 10 KEGG pathways, as well as the top 5 biological processes (BP), molecular functions (MF), and cellular components (CC), for further investigation. We used the online site “SRplot” to visualize the results of the enrichment analysis (Tang et al., 2023), and the KEGG bars was drawn using the bar with color gredient part, the KEGG Sankey diagram was drawn using the sankey dot pathway enrichment part, the GO bubble chart was drawn using the enrichment dot bubble part, the KEGG enrichment circle graph was drawn using the enrichment circle part. To verify the reliability of the key pathways, we performed the pathway enrichment analysis analysis again using Metascape and Funrich.

### 2.7 Acquisition of key pathways of apoptosis

We identified the top-ranked pathways from the KEGG enrichment analysis and selected the genes mapped to these pathways among the common targets. Subsequently, we visualized the differential gene expression results in the TCGA cohort using the “ggplot2” package and mapped these genes to the PI3K-Akt signaling pathway using the KEGG database (Kanehisa et al., 2021). Furthermore, we obtained the human GMT file from the WikiPathways database and utilized the GSEA algorithm to analyze the expression of genes enriched in this pathway, thereby elucidating the overall trend in expression of the PI3K-Akt signaling pathway.

### 2.8 Acquisition of key targets of apoptosis

We utilized Cytoscape software to intersect the Degree value and the MCC algorithm (Chin et al., 2014), along with the differentially expressed genes enriched in the PI3K-Akt signaling pathway, to identify six genes as key targets for subsequent analysis.

### 2.9 Expression and correlation analysis

The online tool UALCAN (Chandrashekar et al., 2022) serves as an information portal for analyzing cancer transcriptome data. In this study, we utilized UALCAN to analyze datasets from the TCGA and CPTAC databases, explored the expression levels of mRNAs and proteins, and conducted survival analysis of key targets related to apoptosis in head and neck squamous cell carcinoma tissues and normal tissues. Additionally, we used the R package “ggcorrplot” to analyze the correlations among the key targets related to apoptosis in the GSE25099 cohort.

### 2.10 Molecular docking

To validate the binding affinity of the active components of FFBZL for apoptosis-related target proteins, molecular docking was conducted for the six active components and the key target proteins associated with apoptosis. The 2D and 3D structures of the active components were retrieved from the PubChem database and saved in SDF format. Subsequently, the 3D structures of the small molecules were energy-minimized using ChemBio3D software and saved in mol2 format. The optimized small molecules were prepared in pdbqt format using AutoDockTools after hydrogenation, charge calculation, charge assignment, and specification of rotatable bonds. The protein structures of the key targets were obtained from the PDB database, processed in PyMOL to remove water molecules and original ligands from the crystal structure, and then imported into AutoDockTools for hydrogenation, charge calculation, charge assignment, and designation of atom types; the protein structures were subsequently saved in pdbqt format. Docking was performed using AutoDock Vina software, and the interaction patterns in the docking results were analyzed using PyMOL software.

## 3 Results

### 3.1 Acquisition of active ingredients of the FFBZL target

**FIGURE 1:**
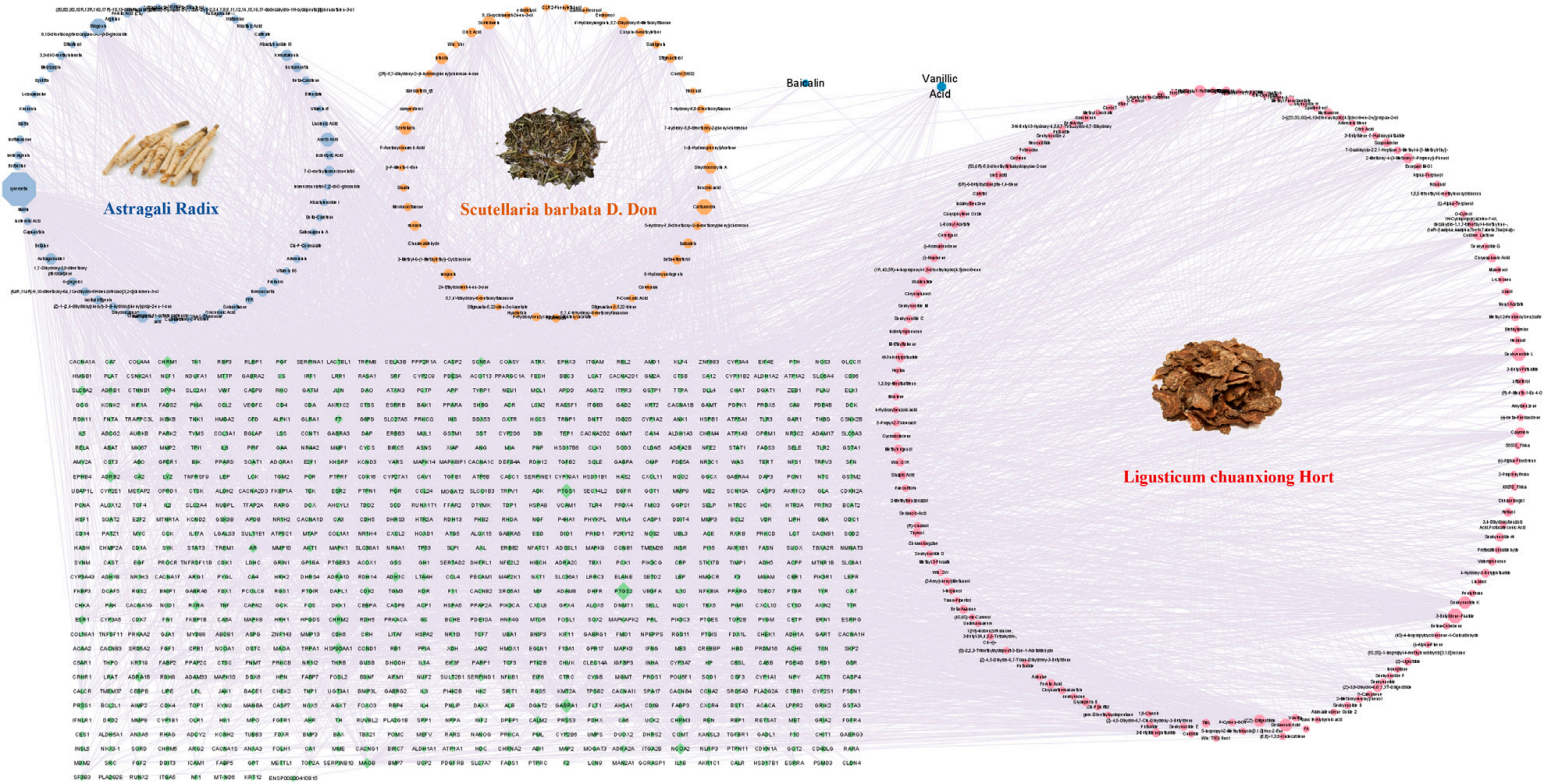
Herb–compound–target network.

The active ingredients of FFBZL were analyzed across multiple databases, resulting in the identification of 61 components of SBD, 73 components of AR, and 154 components of CX. The chemical structures of the components lacking related target information were obtained from PubChem, and the predicted targets were obtained from the PharmMapper database, revealing 349 targets for SBD, 439 targets for AR, and 479 targets for CX. Using Cytoscape software, a “drug-component−target” network for FFBZL was constructed, consisting of a total of 1,088 nodes and 4,235 edges (Figure 1). By evaluating the degree values within this network, six key components of FFBZL were identified: quercetin, wogonin, carthamidin, scutellarein, senkyunolide K, and astragalosidei: astragaloside I (Supplementary Table S1).

### 3.2 Prediction of potential targets of FFBZL in treating OSCC

**FIGURE 2:**
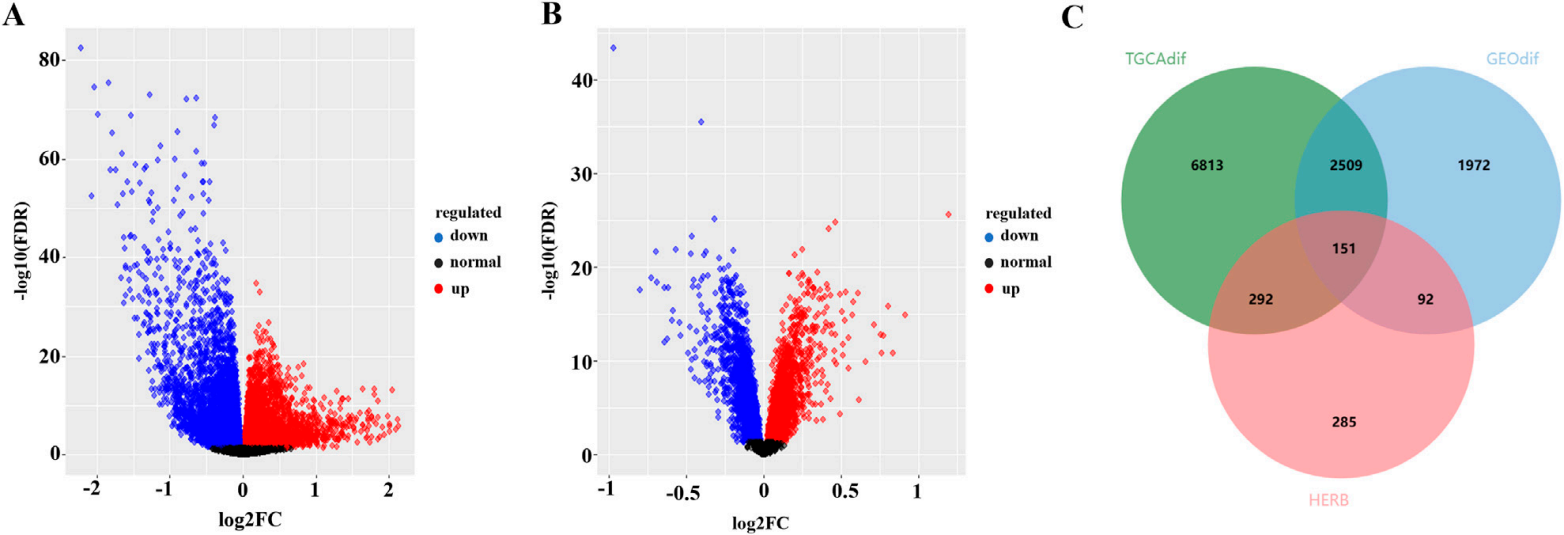
Common target acquisition. **(A)** Volcano plot of DEGs in the TCGA dataset; **(B)** volcano plot of DEGs in the GSE25099 dataset; **(C)** Venn diagram of FFBZL targets with OSCC-related targets.

**FIGURE 3:**
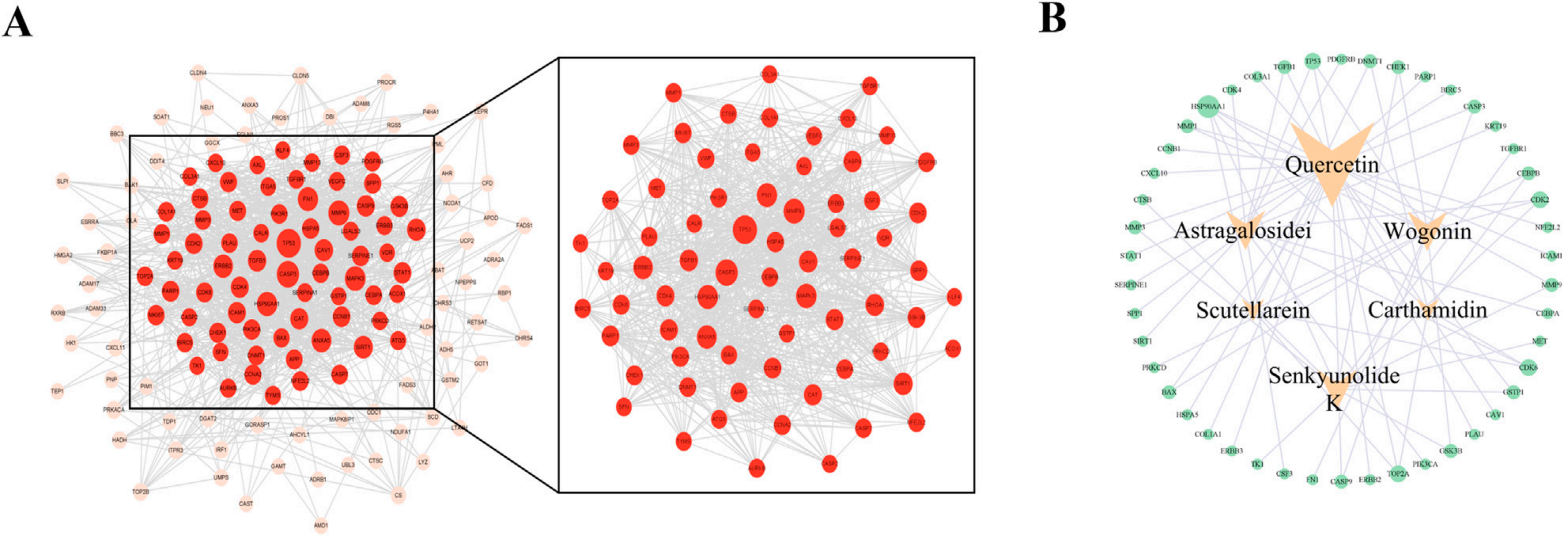
Network analysis. **(A)** PPI network of anti-OSCC targets in FFBZL; **(B)** key component−OSCC−target network.

To identify potential targets for the binding of these active ingredients in OSCC, we downloaded data from the TCGA and GEO databases. The TCGA database contains 9,765 DEGs in OSCC samples, with 5,074 upregulated genes (in red) and 4,691 downregulated genes (in blue) (Figure 2A). The GEO database analysis revealed 4,724 DEGs, 2,255 of which were upregulated (in red) and 2,469 of which were downregulated (in blue) (Figure 2B). The jvenn tool was used to intersect the DEGs between FFBZL and OSCC, which revealed 151 common targets (Figure 2C). These 151 common targets were input into the STRING database to construct a PPI network, which included 143 nodes and 1,129 edges. The network was then imported into Cytoscape for network analysis, and a network section with a degree greater than 12 (the median) was obtained which included 71 nodes and 832 edges (Figure 3A). Using Cytoscape software, a “key component-common target” network of FFBZL was constructed (Figure 3B). In this network, active components such as quercetin, wogonin, astragaloside, and senkyunolide K were directly associated with 27, 11, 11, and 11 of the 71 common targets between FFBZL and OSCC, respectively, this results indicating that these components play crucial roles in the network and are key components in the anti-OSCC effects of FFBZL.

### 3.3 FFBZL induces the apoptosis of OSCC cells through the PI3K-AKT pathway

**FIGURE 4:**
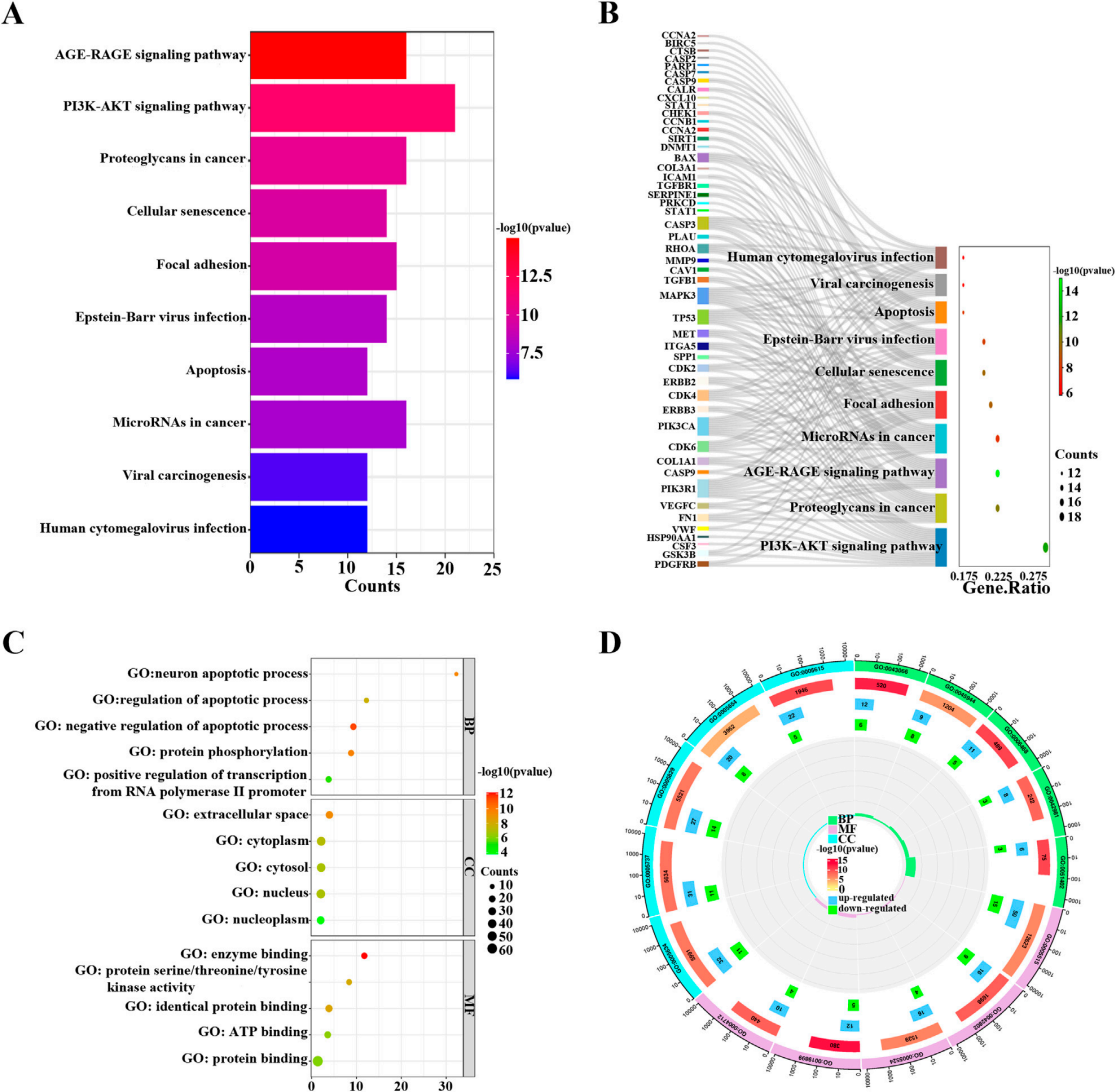
KEGG and GO enrichment analysis **(A)** KEGG bars; **(B)** KEGG Sankey diagram; **(C)** GO bubble chart; **(D)** KEGG enrichment circle graph.

**FIGURE 5:**
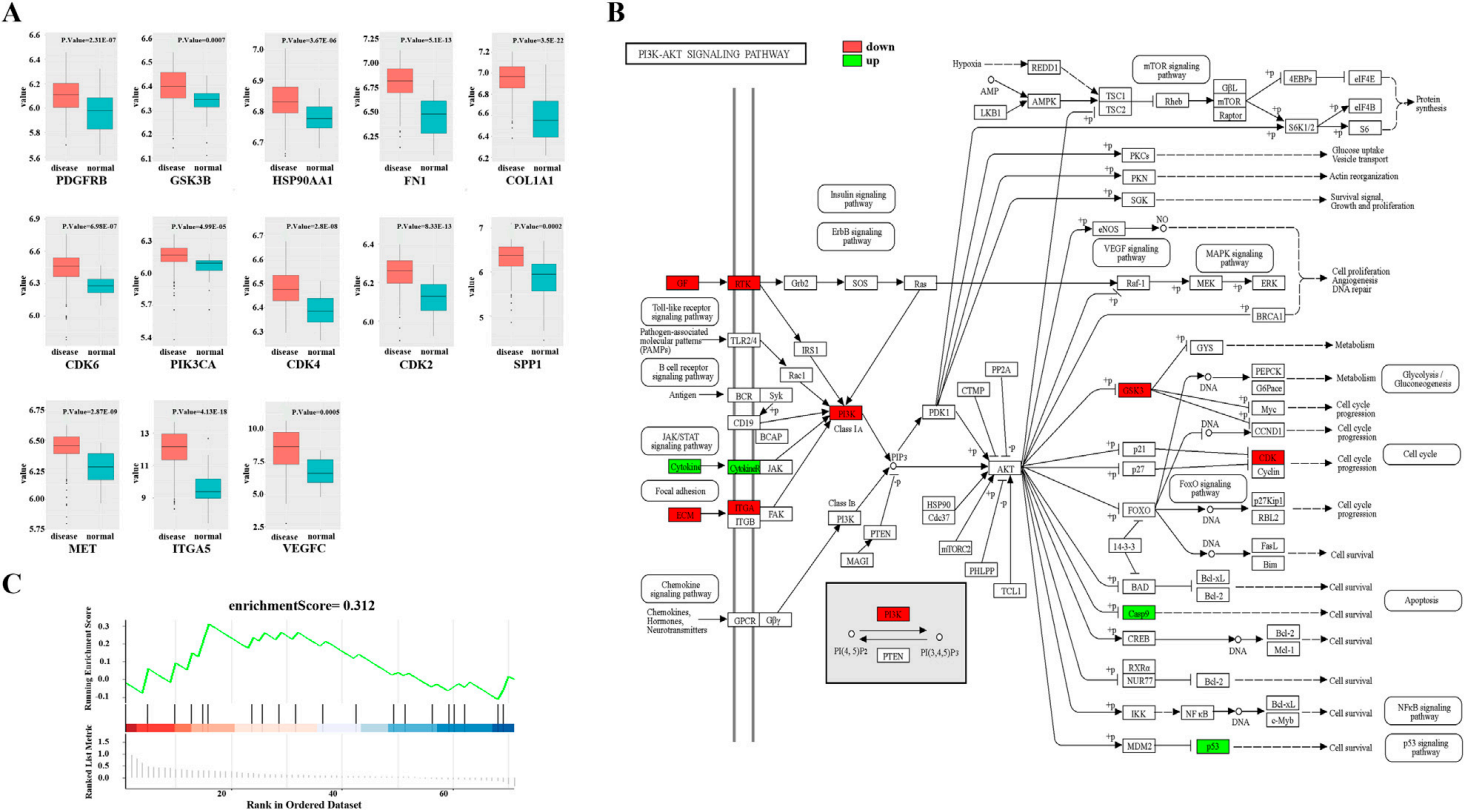
PI3K-Akt signaling pathway. **(A)** Visualization of the expression results of 13 upregulated genes in the TCGA dataset; **(B)** PI3K-Akt signaling pathway in the KEGG dataset; **(C)** GSEA of the expression levels of PI3K-Akt signaling pathway components in normal tissues and OSCC tissues.

To investigate the potential mechanisms of FFBZL effect on OSCC, we conducted KEGG and GO enrichment analyses of FFBZL and 71 common targets in OSCC. The KEGG enrichment analysis revealed that the top 10 pathways were related to cell growth (Supplementary Table S2); the GO results for the top 5 biological processes and molecular functions were also associated with cell growth (Supplementary Table S3). Among the top-ranked biological processes, most were related to apoptosis; these processes included negative regulation of the apoptotic process, regulation of the apoptotic process, and the neuronal apoptotic process (Figure 4). These results suggest that FFBZL may regulate the apoptotic process in OSCC. Therefore, the subsequent investigation focused on the impact of FFBZL on the apoptotic process in OSCC cells. KEGG enrichment analysis highlighted the PI3K-Akt signaling pathway as the top-ranked pathway (Figure 4; Supplementary Figure S3). As 19 genes among the 71 common targets between OSCC and FFBZL were enriched in the PI3K-Akt signaling pathway. According to the results of the differential expression analyses based on the TCGA and GEO, 13 genes, including SPP1, COL1A1, FN1, VEGFC, ITGA5, PDGFRB, MET, CDK6, CDK2, PIK3CA, CDK4, HSP90AA1, and GSK3B, were upregulated (Figure 5A), while 6 genes, including CASP9, VWF, TP53, MAPK3, PIK3R1, and CSF3, were downregulated (Supplementary Figure S1, Supplementary Figure S2). Visualization of the differential expression of the 19 genes from the TCGA data using the “ggplot2” package and mapping of these genes to the PI3K-Akt signaling pathway using the KEGG database were also performed; the upregulated genes are shown in red, and the downregulated genes are shown in green (Figure 5B). Additionally, GSEA of the expression levels of PI3K-Akt signaling pathway components in normal tissues and OSCC tissues revealed an enrichment score of 0.312 for the PI3K-Akt signaling pathway in OSCC, indicating an overall upregulation trend (Figure 5C). Taken together, these findings indicate that the PI3K-Akt signaling pathway plays a crucial role in the proapoptotic effect of FFBZL in OSCC cells.

### 3.4 Screening of key targets involved in the proapoptotic effect of FFBZL on OSCC cells

**FIGURE 6:**
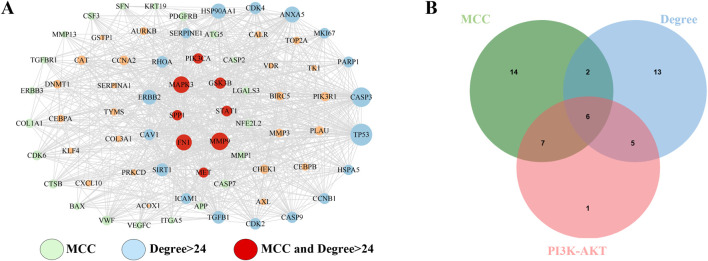
Key target acquisition. **(A)** DEGREE algorithm and MCC algorithm; **(B)** apoptosis-related key targets.

**FIGURE 7:**
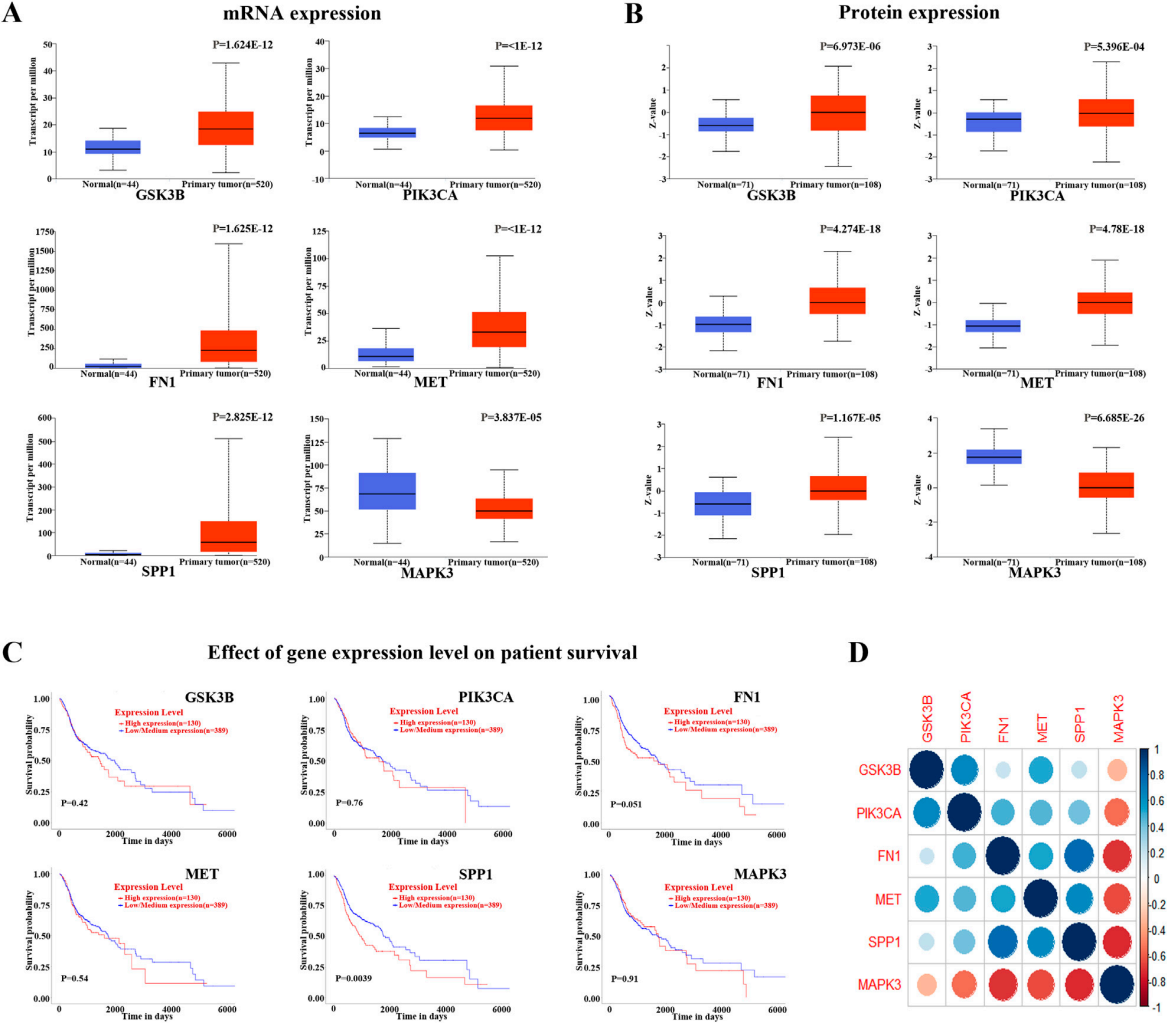
Key target analysis **(A)** mRNA expression analysis of HNSC; **(B)** protein expression analysis of HNSC; **(C)** survival analysis of HNSC; **(D)** correlation analysis between key apoptosis-related targets.

The 71 common targets of FFBZL and OSCC were imported into the STRING database, with the species limited to “*Homo sapiens*,” and a protein–protein interaction network was constructed with a confidence score greater than 0.4. The interacting proteins with a combined score greater than 0.998 included AURKB-BIRC5, CAV1-TGFBR1, CCNA2-CDK2, CDK2-CCNB1, CDK2-TP53, CDK2-CEBPA, CDK4-HSP90AA1, ERBB2-HSP90AA1, ERBB3-PIK3R1, FN1-ITGA5, TP53-GSK3B, HSP90AA1-TP53, PIK3CA-PIK3R1, PLAU-SERPINE1, and SFN-TP53, indicating the importance of these protein interactions in the network. Using Cytoscape software and the Degree and MCC algorithms (Figure 6A), we identified 29 targets via the MCC algorithm and 26 targets with a degree greater than 24; we intersected these genes with the 19 DEGs enriched in the PI3K-Akt signaling pathway (Figure 6B) to reveal 6 key targets: GSK3B, PIK3CA, FN1, MET, SPP1, and MAPK3. Among these genes, GSK3B, PIK3CA, FN1, MET, and SPP1 were upregulated, while MAPK3 was downregulated. These 6 genes were selected as key targets for further analysis. To explore the clinical significance of the key targets related to apoptosis, we used the UALCAN online tool for expression analysis. The results showed that the mRNA (Figure 7A) and protein (Figure 7B) expression of GSK3B, PIK3CA, FN1, MET, and SPP1 was significantly upregulated in head and neck squamous cell carcinoma tissues, while MAPK3 was significantly downregulated. Survival analysis revealed that patients with low expression of MET, SPP1, FN1, and MAPK3 had increased survival times, with significant increases observed in patients with low expression of SPP1 and FN1 (Figure 7C). The analysis of correlations revealed significant positive correlations among GSK3B, PIK3CA, FN1, MET, and SPP1 (Figure 7D), suggesting potential synergistic interactions among these 5 upregulated key targets related to apoptosis.

### 3.5 Interactions between key components of FFBZL and apoptosis targets in OSCC

**FIGURE 8:**
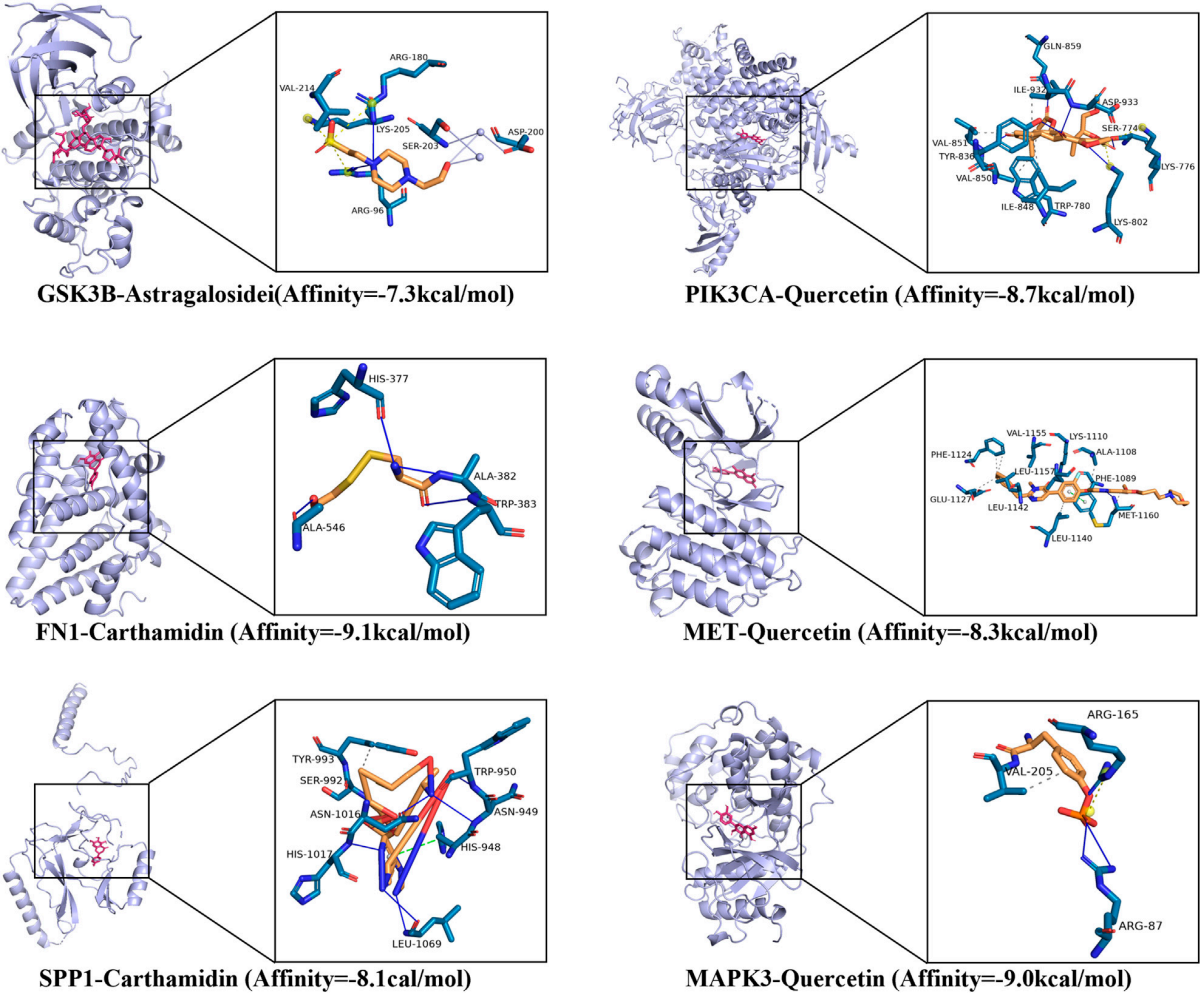
Molecular docking visualization results.

Molecular docking analysis revealed that the six active components of FFBZL, quercetin, wogonin, carthamidin, scutellarein, senkyunolide K and astragalosidei: astragaloside I, have good binding potential with the following predicted apoptosis targets: GSK3B, PIK3CA, FN1, MET, and SPP1 (Supplementary Table S4). A binding energy less than −5 kcal/mol indicates a good binding effect. Among them, the strongest binding potential was observed for carthamidin binding to the FN1 protein, with a binding energy of −9.1 kcal/mol; the formation of hydrogen bonds with GLU, ARG, and HIS, with hydrogen bond lengths of 1.42 Å, 2.65 Å, and 2.29 Å; and hydrophobic interactions with LEU and ALA. The molecular docking results of the components with the target proteins were visualized (Figure 8), which demonstrated good binding. These results confirmed that the treatment of OSCC with FFBZL is achieved through the binding of key components to apoptosis targets.

## 4 Discussion

Oral cancer is one of the most common malignant tumors in the field of oral and maxillofacial surgery. Conventional treatment methods such as surgery, radiotherapy, and chemotherapy have limited efficacy and are accompanied by varying degrees of adverse reactions, suggesting the need for alternative treatment modalities to alleviate these issues. Studies have shown that FFBZL has good therapeutic efficacy against oral cancer. However, the specific effective components and the involved antitumor mechanism of FFBZL have yet to be explored. Therefore, in this study, we theoretically predicted the core components, targets, and pathways of FFBZL involved in its anti-OSCC effects through network pharmacology and bioinformatics analyses and subsequently validated these predictions through molecular docking.

The analysis of the core anti-OSCC effects of FFBZL revealed that quercetin, wogonin, carthamidin, scutellarein, senkyunolide K, and astragaloside are key components. Wogonin and scutellarein are both flavonoids, and experimental evidence has shown that flavonoids and phenols are inhibitors of the initiation stage of cancer; they regulate biological signal transduction, leading to the upregulation of proapoptotic protein expression; moreover, by controlling the growth of vascular endothelial cells, they block the formation of blood vessels in tumor tissues, thereby inhibiting solid tumor growth (Wen et al., 2021). Quercetin can inhibit the growth, proliferation, invasion, and migration of various tumor cells and induce apoptosis (Tang et al., 2020). Carthamidin B is an alkaloid with tetrahydro-β-carboline as its parent structure that has tumor-suppressive effects in rectal cancer and breast cancer (Luo et al., 2015; Wang et al., 2021). Senkyunolide compounds can inhibit the generation of reactive oxygen species (ROS) and induce antioxidative damage through apoptosis (Qi et al., 2010). Astragalosidei can induce the apoptosis of various tumor cells by decreasing the expression level of BCL-2 and increasing the expression level of BAX (Wang et al., 2023). The antitumor effects of the core components of the drug are mostly related to apoptosis, indicating that the anti-OSCC effects of FFBZL may be related to the apoptosis of oral cancer cells.

Cell apoptosis is one of the intrinsic mechanisms for regulation and maintenance of the cell cycle and is capable of eliminating nonfunctional, harmful, and abnormal cells in a timely manner (Carneiro and El-Deiry, 2020). For a long time, cell apoptosis has been considered an important mechanism for preventing the occurrence of tumors (Hanahan and Weinberg, 2011). Through GO functional and KEGG pathway enrichment analyses of the core components of FFBZL and the common targets in OSCC, this study revealed that FFBZL acts on pathways related to cell growth and apoptosis, with the PI3K-Akt pathway playing a major role. The PI3K-Akt pathway is a common signaling pathway that plays an important regulatory role in various aspects of cell proliferation, survival, and metabolism. The PI3K/Akt signaling pathway can inhibit cell apoptosis (Li et al., 2023), prevent programmed cell death in tumor cells, and promote tumor cell proliferation and survival (Fan et al., 2022). Among the 71 common targets of OSCC and FFBZL, 21 genes were found to be enriched in the PI3K-Akt signaling pathway, with 13 genes exhibiting high expression in OSCC. Additionally, the KEGG database was used to map the 21 genes to the PI3K-Akt signaling pathway, which demonstrated an overall upregulation trend of genes in the PI3K-Akt signaling pathway in OSCC. Furthermore, GSEA of the PI3K-Akt signaling pathway in OSCC yielded an enrichment score of 0.312, which also indicated an upregulation trend for components of this pathway. Overall, FFBZL may influence the apoptosis of OSCC cells through the PI3K-Akt signaling pathway.

The analysis in this study identified six key targets through which FFBZL influences apoptosis in OSCC cells, namely, GSK3B, PIK3CA, FN1, MET, SPP1, and MAPK3. GSK3B is expressed as glycogen synthase kinase 3β (GSK-3β), a critical inhibitor of the Wnt/β-catenin signaling pathway, and is generally suppressed in tumor cells (Zhu et al., 2022). Mishra et al. reported that overexpression of GSK3B plays a greater role in oral squamous cell carcinoma (OSCC) than in other cancers due to the activation of the downstream transcription factor GSK3B, which promotes uncontrolled cell division in OSCC, suggesting a link between the occurrence of OSCC and increased GSK3B expression (Mishra et al., 2015). PIK3CA encodes the p110α catalytic subunit of phosphatidylinositol 3-kinase (PI3K), which plays a significant role in regulating important functions, such as cell proliferation, metabolism, protein synthesis, angiogenesis, and cell apoptosis, through its role in the PI3K/Akt pathway (Lai et al., 2015). Fibronectin 1 (FN1) is a widely distributed glycoprotein in the extracellular matrix that plays a crucial role in cancer onset and development (Wang et al., 2022). Huang et al. reported a positive correlation between PI3K-Akt signaling pathway activity and PIK3CA and FN1 expression (Huang et al., 2022). MET is an oncogene that encodes the transmembrane receptor protein MET with tyrosine kinase activity and participates in the occurrence and development of various human cancers and mediates proliferation, migration, and invasion (Yang X. et al., 2022). Studies have shown that MET is closely related to PI3K-Akt signaling, as activated MET further activates PI3K to subsequently activate Akt, which regulates cell survival, proliferation, and metabolism, promoting cell growth and inhibiting apoptosis (Bian et al., 2018). SPP1 is a key gene in head and neck cancer, and its expression is closely related to the infiltration of immune cells, especially M2 macrophages, in head and neck cancer. Shunli Feng et al. demonstrated that silencing SPP1 leads to a decrease in invasion and metastasis in head and neck cancer (Feng et al., 2022). Furthermore, some studies suggest that the upregulation of SPP1 may be related to the activation of the PI3K/AKT pathway. Overexpression of SPP1 in tumor cells may promote activation of the PI3K/AKT signaling pathway, thereby enhancing tumor cell proliferation and invasion (Zhang et al., 2023). Through multiple cascading phosphorylation events, MAPK3 ultimately regulates processes such as cell growth, proliferation, differentiation, and survival by phosphorylating downstream proteins (Taherkhani et al., 2023). The protein encoded by MAPK3 is a member of the MAP kinase family, also known as extracellular signal-regulated kinase (ERK), which acts in a signaling cascade to regulate various cell processes in response to various extracellular signals (Deng et al., 2021). Notably, survival analysis of OSCC cells with respect to the six apoptosis target genes affected by FFBZL showed that high expression of FN1 and SPP1 is associated with a poor patient prognosis. In conclusion, when GSK3B, PIK3CA, FN1, MET, SPP1, and MAPK3 are overexpressed, the PI3K-Akt pathway is activated, inhibiting apoptosis. The results of the present study suggested that FFBZL may exert its antitumor effects by downregulating the expression of these six key targets, which subsequently modulates the PI3K-Akt pathway to activate apoptosis, ultimately extending patient survival.

## 5 Limitations

In this study, a preliminary attempt was made to predict the effective active compounds, their expected targets and associated pathways for the treatment of oral squamous carcinoma by compound hemicrania, thus providing theoretical evidence for future experimental and clinical studies required for a thorough investigation of the potential application of compound hemicrania as an anti-oral squamous carcinoma drug. Since web pharmacology relies on data mining from different databases, it has inherent limitations and needs to be validated by clinical trials.

## 6 Conclusion

**FIGURE 9:**
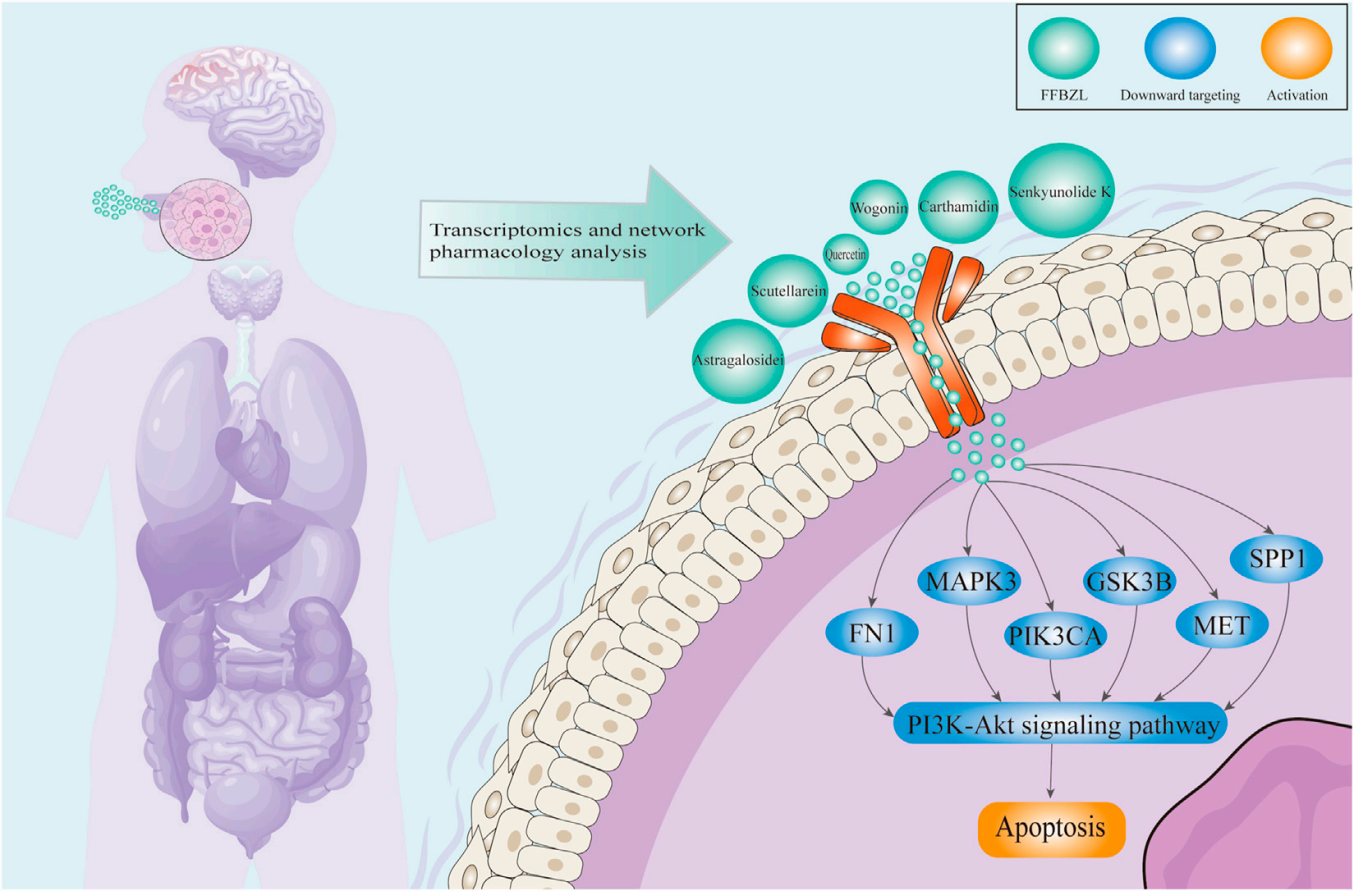
Schematic illustration of the mechanism of FFBZL in the treatment of OSCC.

This study comprehensively explored the potential mechanisms of FFBZL in the treatment of OSCC using traditional Chinese medicine network pharmacology, bioinformatics, and molecular docking approaches. The results indicate that FFBZL may effectively bind to apoptotic targets through its active components; downregulate the expression of the key apoptosis targets GSK3B, PIK3CA, FN1, MET, SPP1, and MAPK3; and regulate the PI3K-Akt pathway, promoting tumor cell apoptosis to exert antitumor effects and thereby improving the prognosis of OSCC patients (Figure 9). These findings provide new insights into the mechanism of action of FFBZL in the treatment of OSCC, providing a theoretical foundation and scientific basis for its clinical application.

## Data Availability

The original contributions presented in the study are included in the article/Supplementary Material, further inquiries can be directed to the corresponding authors.
