# Supplementary figures and images for: Network pharmacology and transcriptomics reveal the mechanisms of FFBZL in the treatment of oral squamous cell carcinoma
Source: Front Pharmacol. 2024 Sep 11;15:1405596. doi: 10.3389/fphar.2024.1405596 (PMC11422709; doi:10.3389/fphar.2024.1405596)

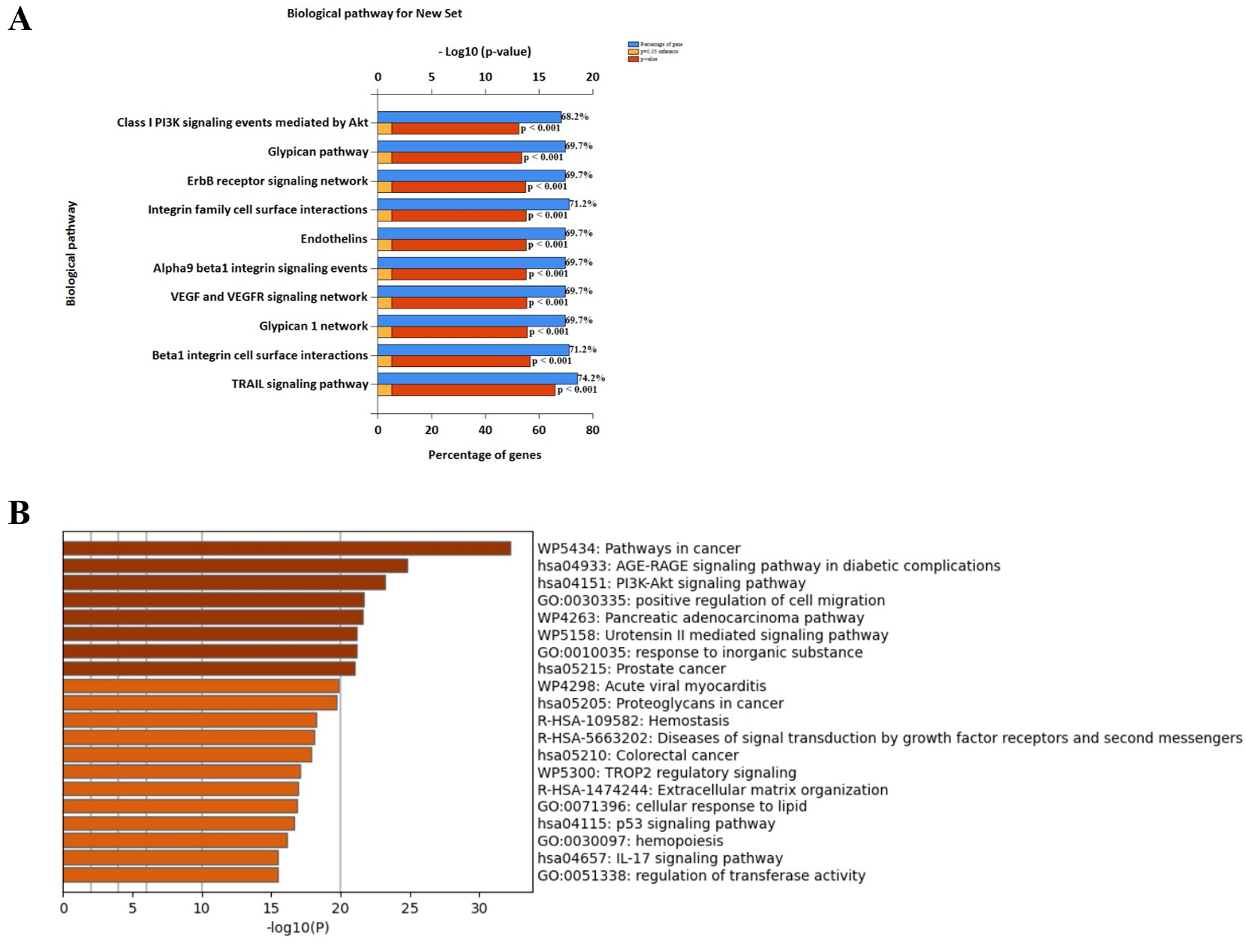

Supplement: Supplementary file 1 [file Image3.tif]

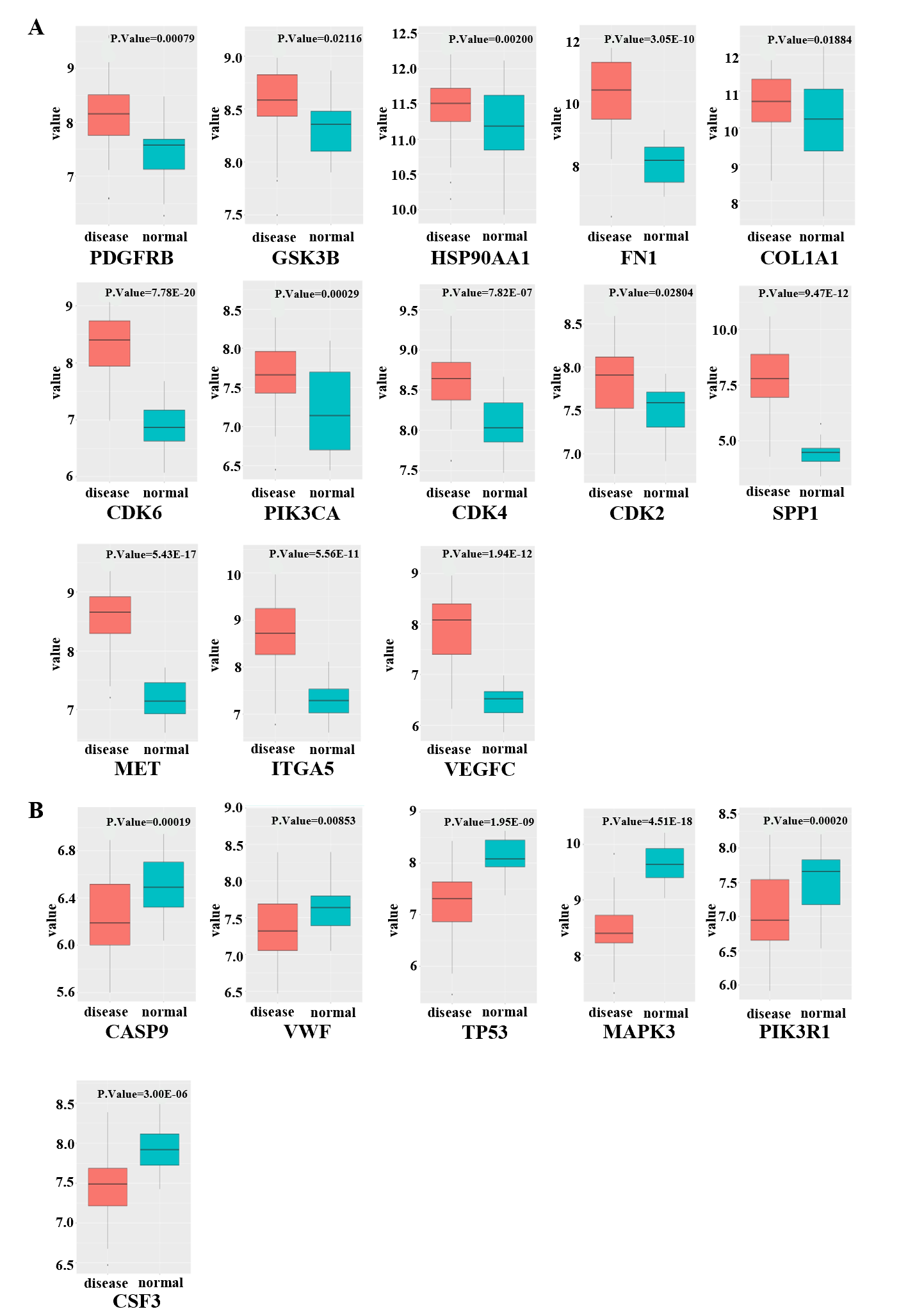

Supplement: Supplementary file 2 [file Image2.tif]

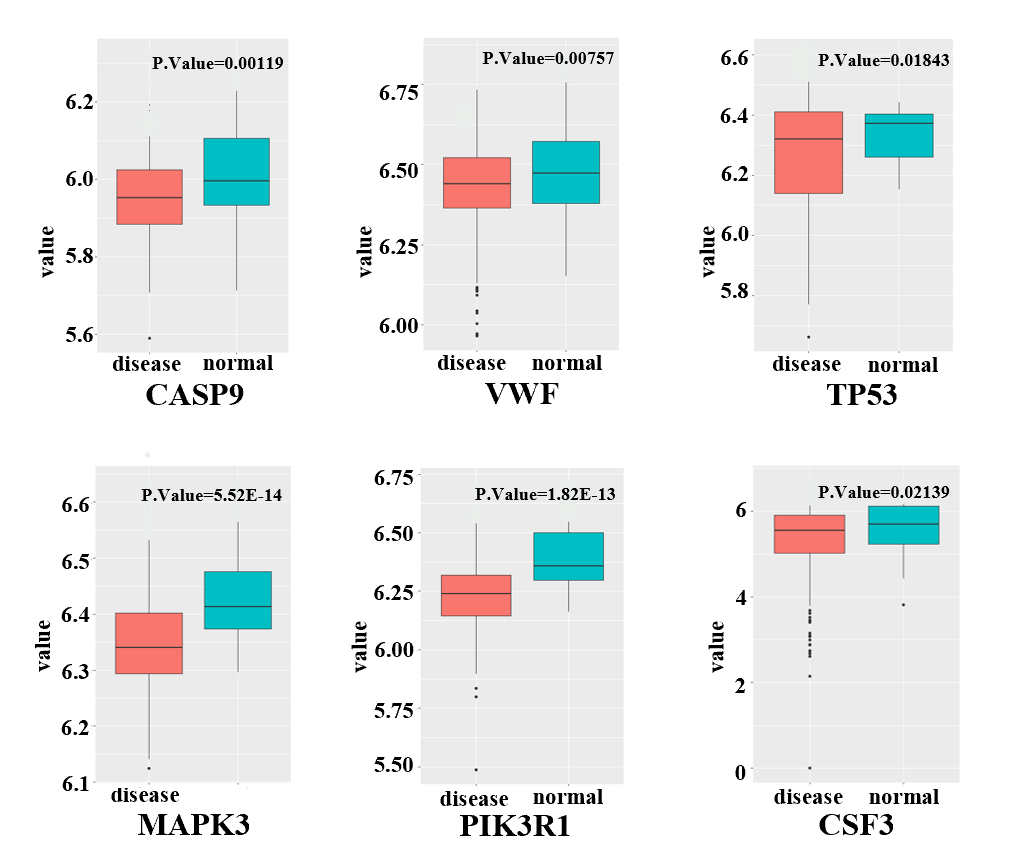

Supplement: Supplementary file 3 [file Image1.tif]
