# Supplementary material for: Network pharmacology and transcriptomics reveal the mechanisms of FFBZL in the treatment of oral squamous cell carcinoma
Source: Front Pharmacol. 2024 Sep 11;15:1405596. doi: 10.3389/fphar.2024.1405596 (PMC11422709; doi:10.3389/fphar.2024.1405596)
Supplement: Supplementary file 4 [file DataSheet1.pdf]

Supplementary materials

Name	Molecular formula	Molecular mass	Number of hydrogen bond donors	Number of hydrogen bond receptors	Lipid-water partition coefficient	Rotatable key	Degree
Quercetin	C15H10O7	302.23	5	7	1.5	1	292
Wogonin	C16H12O5	284.26	2	5	3	2	96
Carthamidin	C15H12O6	285.25	4	6	2	1	93
Scutellarein	C15H10O6	286.24	4	6	1.4	1	72
Senkyunolide K	C12H16O3	208.25	1	3	1.6	3	64
Astragalosidei	C45H72O16	869	7	16	2.4	11	52

Table S1 6 key Ingredients in FFBZL.

KEGG	Genetic Ratio	Ratio/%	PValue	FDR
hsa04151:PI3K-Akt signaling pathway	21/71	29	1.94E-12	5.05E-11
hsa05205:Proteoglycans in cancer	16/71	22	4.83E-11	7.18E-10
hsa04933:AGE-RAGE signaling pathway	16/71	22	1.03E-15	5.34E-14
hsa05206:MicroRNAs in cancer	16/71	22	1.53E-08	7.22E-08
hsa04510:Focal adhesion	15/71	21	4.5E-10	3.6E-09
hsa04218:Cellular senescence	14/71	20	2.2E-10	2.21E-09
hsa05169:Epstein-Barr virus infection	14/71	20	5.39E-09	3.11E-08
hsa04210:Apoptosis	12/71	17	8.54E-09	4.44E-08
hsa05203:Viral carcinogenesis	12/71	17	5.56E-07	1.75E-06
hsa05163:Human cytomegalovirus infection	12/71	17	1.47E-06	4.13E-06

Table S2 KEGG enrichment analysis.

Number	GO	type	Genetic Ratio	Ratio/%	PValue	FDR
GO:0043066	negative regulation of apoptotic process	BP	18/71	25	4.14E-12	6.48E-09
GO:0045944	positive regulation of transcription from RNA polymerase II promoter	BP	17/71	24	5.63E-06	0.000315
GO:0006468	protein phosphorylation	BP	16/71	22	2.2E-10	8.61E-08
GO:0042981	regulation of apoptotic process	BP	11/71	15	1.83E-08	3.19E-06
GO:0051402	neuron apoptotic process	BP	9/71	13	3.03E-10	9.49E-08
GO:0005515	protein binding	MF	66/71	93	8.74E-07	3.05E-05
GO:0042802	identical protein binding	MF	25/71	35	5.32E-09	5.83E-07
GO:0005524	ATP binding	MF	21/71	30	5.7E-07	2.27E-05
GO:0019899	enzyme binding	MF	17/71	24	6.03E-13	1.68E-10
GO:0004712	protein serine/threonine/tyrosine kinase activity	MF	14/71	20	8.36E-09	5.83E-07
GO:0005634	nucleus	CC	43/71	61	1.27E-07	4.57E-06
GO:0005737	cytoplasm	CC	42/71	60	7.09E-08	4.06E-06
GO:0005829	cytosol	CC	41/71	58	1.42E-07	4.57E-06
GO:0005654	nucleoplasm	CC	28/71	39	0.000176	0.002334
GO:0005615	extracellular space	CC	27/71	38	5.1E-10	1.15E-07

Table S3 GO enrichment analysis.

Protein Name	Test Compounds	Affinity/kcal·mol-1
GSK3B	Astragalosidei	-7.3
GSK3B	Carthamidin	-6.2
GSK3B	Quercetin	-6.6
GSK3B	Scutellarein	-6.2
GSK3B	Senkyunolide K	-5.4
GSK3B	Wogonin	-6.4
PIK3CA	Astragalosidei	-5.4
PIK3CA	Carthamidin	-8.5
PIK3CA	Quercetin	-8.7
PIK3CA	Scutellarein	-8.5
PIK3CA	Senkyunolide K	-6.4
PIK3CA	Wogonin	-8
FN1	Carthamidin	-9.1
FN1	Quercetin	-8.7
FN1	Scutellarein	-9
FN1	Senkyunolide K	-7.5
FN1	Wogonin	-8.3
MET	Astragalosidei	-6.7
MET	Carthamidin	-8.3
MET	Quercetin	-8.7
MET	Scutellarein	-8.5
MET	Senkyunolide K	-6.5
MET	Wogonin	-8.2
SPP1	Astragalosidei	-6.1
SPP1	Carthamidin	-8.1
SPP1	Quercetin	-7.7
SPP1	Scutellarein	-7.6
SPP1	Senkyunolide K	-7.3
SPP1	Wogonin	-7.1
MAPK3	Carthamidin	-8.7
MAPK3	Quercetin	-9.0
MAPK3	Scutellarein	-8.5
MAPK3	Senkyunolide K	-6.4
MAPK3	Wogonin	-8.1

Table S4 molecular docking.

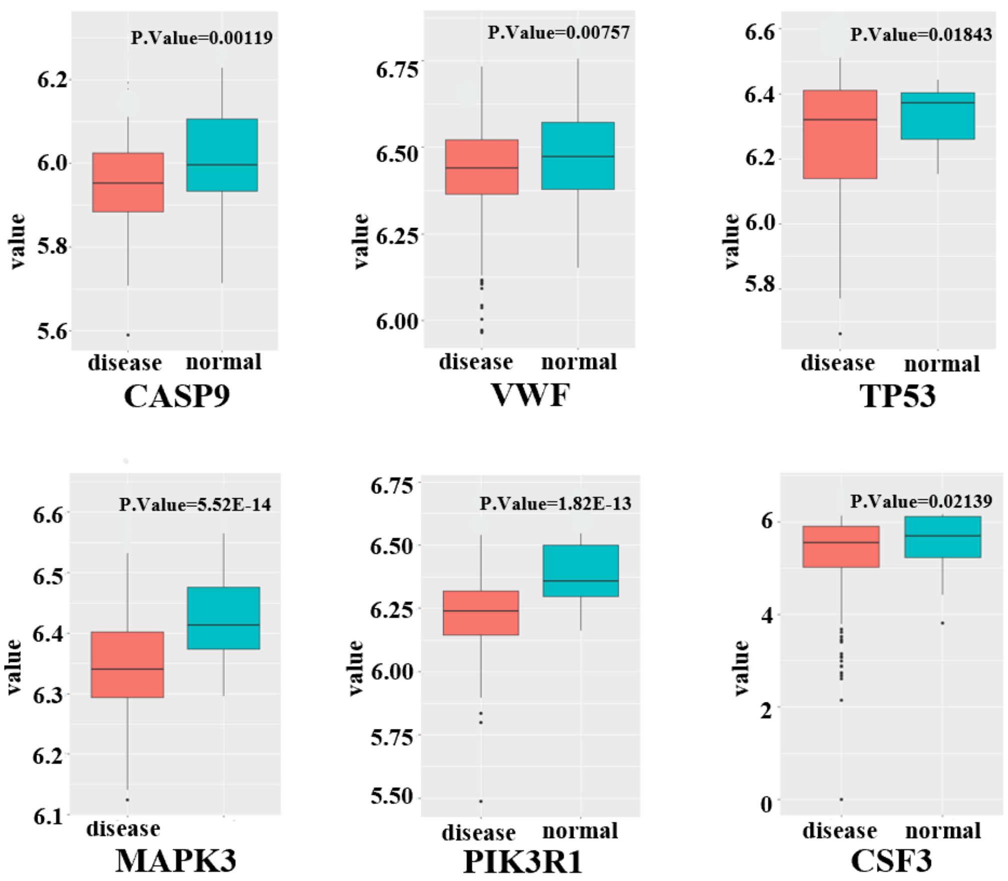

FIGURE S1 6 down-regulated genes in the TCGA dataset.

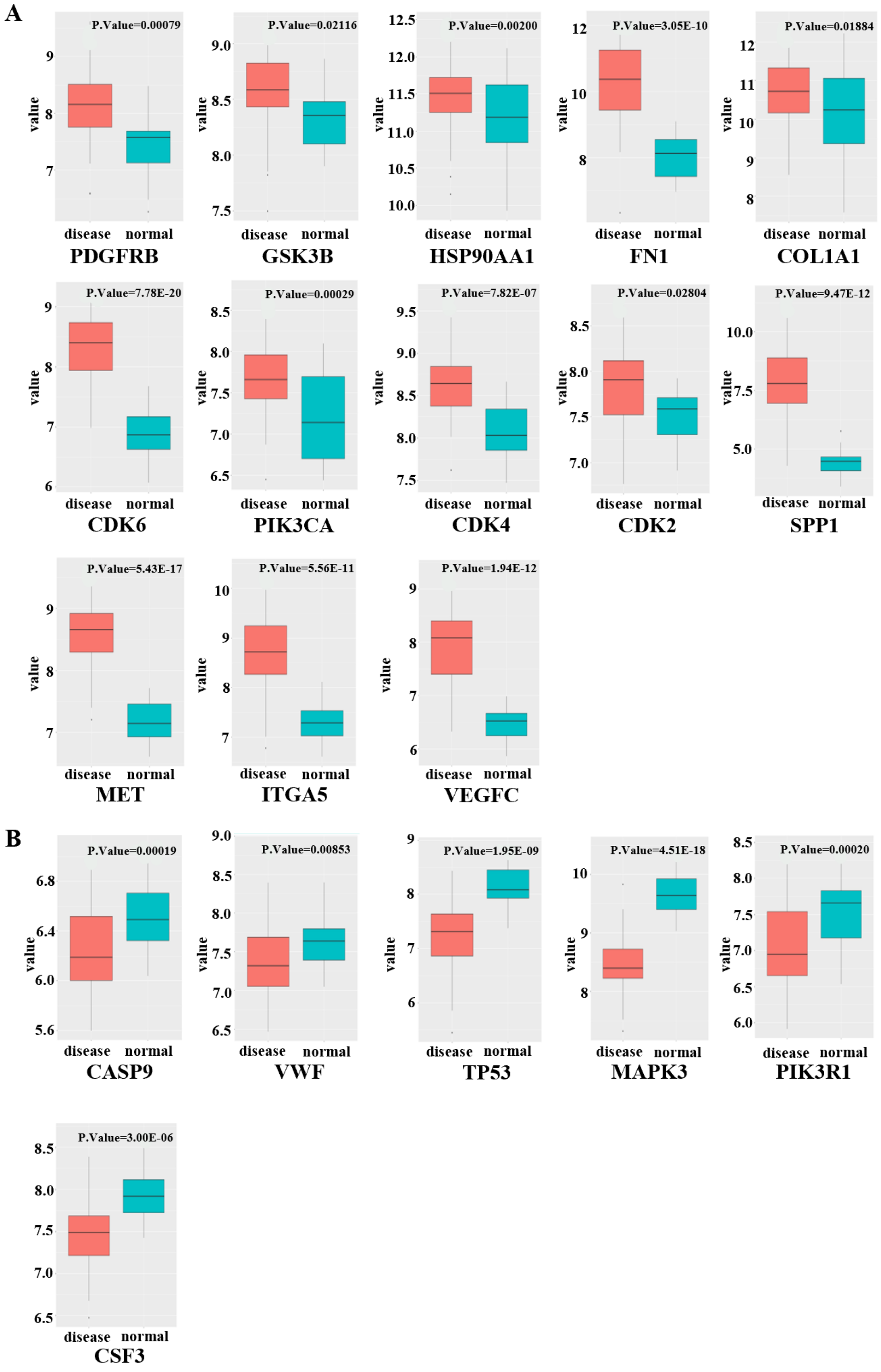

FIGURE S2 Visualization of the expression results of 19 genes in the GEO dataset. (A) 13 up-regulated genes in the GEO dataset; (B) 6 down-regulated genes in the GEO dataset.

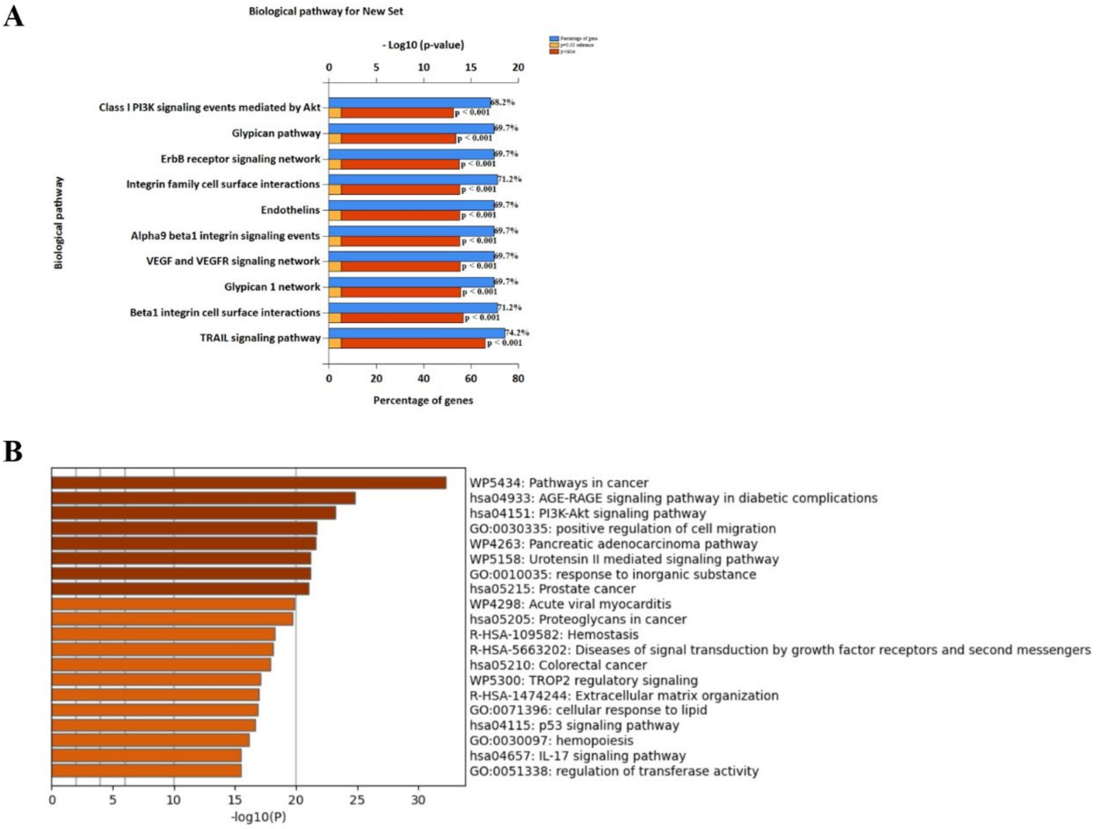

FIGURE S3 Kegg enrichment analysis results. (A) Funrich kegg enrichment analysis results; (B) Metascape kegg enrichment analysis results.
